# Spatiotemporal all-optical diffractive neural networks empowered by spatiotemporal light field manipulation

**DOI:** 10.1038/s41377-026-02366-7

**Published:** 2026-09-16

**Authors:** Fu Feng, Ziyang Zhang, Dewang Huo, Xiaolong Li, Qinggang Lin, Xingyu Zhao, Guozhong Hou, Daoxin Dai, Michael G. Somekh, Xiaocong Yuan

**Affiliations:** 1 Research Center for Frontier Fundamental Studies, Zhejiang Lab, Hangzhou, 311100 China; 2 State Key Laboratory of Extreme Photonics and Instrumentation, College of Optical Science and Engineering, Zhejiang University, Hangzhou, 310027 China; 3 Zhejiang Key Laboratory of 3D Micro/Nano Fabrication and Characterization, Westlake Institute for Optoelectronics, Hangzhou, Zhejiang 311421 China; 4 College of Optical and Electronic Technology, China Jiliang University, Hangzhou, Zhejiang 310018 China; 5 Faculty of Engineering, University of Nottingham, Nottingham, NG7 2RD UK; 6 Key Laboratory of Optoelectronic Devices and Systems of Ministry of Education and Guangdong Province, College of Physics and Optoelectronic Engineering, Shenzhen University, Shenzhen, 518060 China

**Keywords:** Applied optics, Optical techniques

## Abstract

All-optical neural networks (AONNs) offer significant potential for feature detection and pattern recognition by leveraging light-speed processing, high parallelism, and low energy consumption. However, traditional AONNs are limited to spatial light intensity distributions for both inputs and outputs, restricting network learning to the spatial-only dimension. To overcome this, we propose a novel spatiotemporal AONN architecture that integrates the time dimension with the spatial domain, enabling simultaneous modulation of light field information across both time and space. In this configuration, the output spatiotemporal light field is spatially filtered, and its temporal signals are detected using a single-pixel detector. This approach integrates the temporal dimension as a core computational resource into the network, driving a paradigm shift in AONNs from “imaging detection” to “temporal analysis”, effectively overcoming the speed bottleneck caused by the bandwidth limitations of traditional 2D array detectors. Moreover, experimental studies demonstrate that the system can accelerate the processing of real-world spatiotemporal lidar signals, facilitating the real-time reconstruction of multi-target moving scenes. Our innovative spatiotemporal AONN design offers enhanced flexibility, faster processing speeds, and superior performance, paving the way for more efficient and effective optical signal processing and computing applications.

## Abstract (web summary)

Spatiotemporal all-optical neural network (ST-AONN) system. This work incorporates the time dimension into traditional space-only diffractive neural networks, enabling spatiotemporal multi-dimensional light field modulation and signal processing, delivering additional modulation dimensions and faster operational speed.

## Graphical abstract

**Figure:**
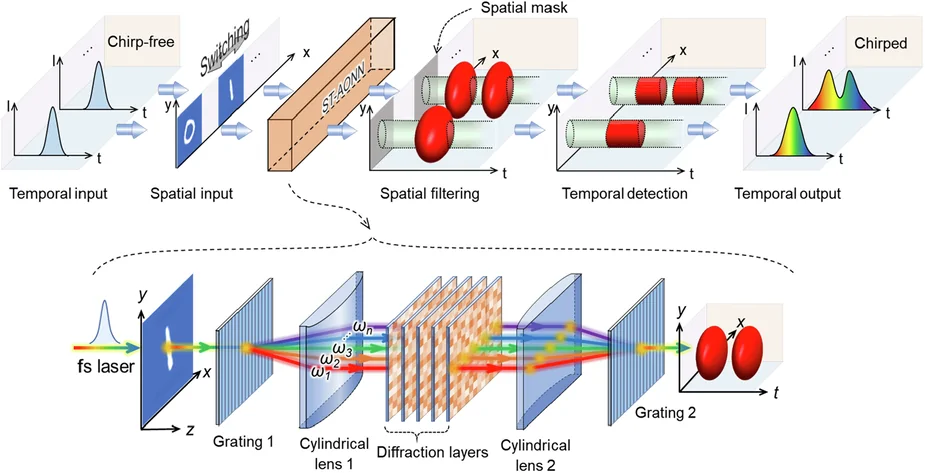

## Introduction

Optical neural networks, as a new type of artificial neural network implementation, can be seen as a statistical data fitter that learns features from large amounts of labeled data through algorithms and summarizes the general mapping relation between input and output spaces^1–4^. These networks use photons as computational carriers, thus possessing advantages such as high parallelism, high throughput, light-speed processing, and low power consumption^5–11^. Demonstrations to date have shown that optical neural networks can fulfill complex computing needs, including feature detection^12–15^, object classification^12,16–22^, speech recognition^23,24^, optical element design^25–28^, material synthesis^29^, among others.

Currently, all-optical neural networks, especially most diffractive neural networks in free space, tend to operate by mapping an input spatial intensity distribution onto a predefined output spatial intensity distribution. This process is often followed by electronic pattern recognition for decision-making. In essence, the network can be viewed as an optical system that performs a space-to-space projection for data processing^12,15,30,31^. This design presents two notable limitations. Firstly, the modulation domain of the network is confined solely to the space, which makes it difficult to process information encoded in other domains. Secondly, the detection of the spatial light intensity necessitates the use of two-dimensional array imaging devices, such as charge-coupled devices (CCD) or complementary metal-oxide-semiconductor (CMOS) sensors, which complicates the integration and scalability of the system. Furthermore, although the calculation of light within a diffraction neural network is performed at the speed of light, the low readout speed from array imaging devices to the memory inherently limits the detection speed, thereby limiting the overall operating speed of the network, typically a few hundred hertz. Adding degrees of freedom to improve the modulation capability of the network as well as enhancing the network’s operation speed remains a significant challenge^32–34^. In recent years, several studies have sought to address these problems. Liu et al.^35^ introduced an all-fiber, single-pixel detection method for image acquisition, using multimode fiber dispersion to convert input images into sub-pulse information with distinct patterns. This approach enables space-to-time information projection and achieves high-speed detection with a single-pixel photodiode (PD); however, the computation itself is performed by electrical neural networks instead of optical ones, which reduces the advantages of optical computing. Zhou et al.^36^ developed a high-speed photonic computing architecture for ultrafast dynamic machine vision that combines digital micromirror devices (DMDs), optical fibers, and active optoelectronic devices to process light fields’ spatial and temporal information. The combination of the spatial modulation process via DMD and photoelectric conversion during temporal modulation results in a rather complex system and poses issues in respect of system scalability.

In addition, Zhang et al.^28^ proposed a space-to-time information projection method based on output aperture-confined intensity detection, directly mapping 2D spatial information to 1D intensity values. Zhou et al.^37^ demonstrated a multi-layer spatiotemporal architecture implemented by cascading multiple pulse shaping systems. And Feng et al.^38^ have recently developed a spatiotemporal mode multiplexing scheme based on diffractive neural networks. However, these works lack direct temporal modulation, passively projecting spatial information into the time domain rather than utilizing time as a core computational resource. For on-chip optical computing, Hamerly et al.^39^ proposed a novel on-chip interconnected photon accelerator that enables high-speed time-domain optical calculations at the GHz level. Similarly, Li et al.^40^ used an end-to-end all-optical recurrent neural network to further increase the processing speed of optical temporal signals to 100 GHz. At the moment, these approaches can only process signals at time domain due to the on-chip architecture, which requires additional pre- or post-processing for handling spatial information.

**Fig. 1:**
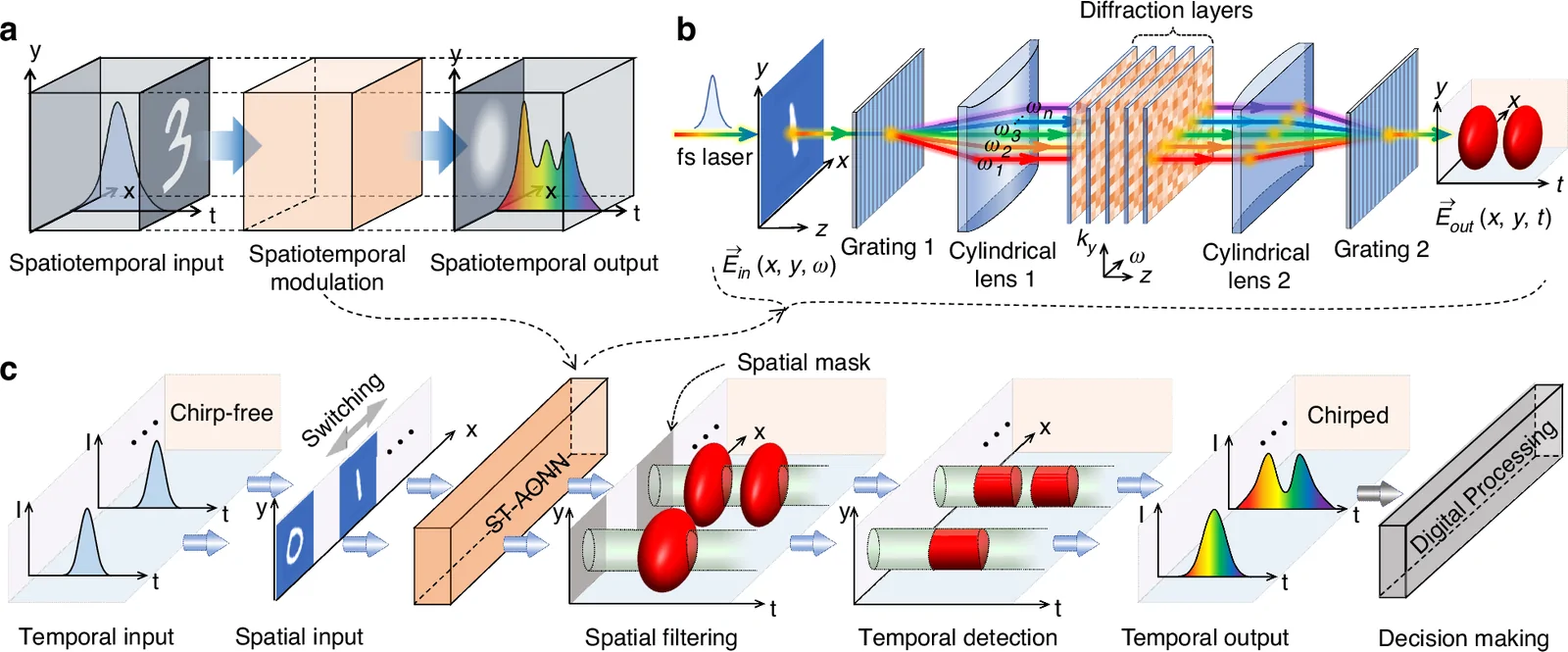
Principle and structure of the ST-AONN system. **a** Schematic diagram of ST-AONN. **b** Structural diagram of ST-AONN. The spatial input is in the *x*-*y* plane, and the light propagates along the *z*-axis. The output light field is in the spatiotemporal domain (*x*-*y*-*t* space). **c** Schematic diagram of the spatiotemporal light field propagation of the ST-AONN system. ST-AONN projects chirp-free pulse light fields with spatial intensity distribution into spatiotemporal structured light fields, and after spatial filtering and temporal detection, temporal outputs can be obtained. The spatial filtering is realized by the spatial mask on the *x*-*y* plane, and the temporal detection is realized based on the single-pixel PD

While previous works have explored projecting spatial information into the temporal domain to facilitate detection and enhance overall system speed, in contrast, this manuscript proposes a novel spatiotemporal all-optical neural network (ST-AONN) that offers additional modulation dimensions and a faster operating speed compared to conventional ONNs limited to the spatial dimension. This new architecture incorporates the time domain in addition to the space domain, enabling two-dimensional modulation in both space and time domains, as shown in Fig. 1a. More precisely, the system operates by first converting the incident light fields, which contain spatial and temporal features, into spatiotemporal structured light fields through spatiotemporal light field modulation. A spatial mask is incorporated into the system to filter the structured light field spatially; the temporal intensity variations of the light passing through the mask serve as the system’s output and are directly measurable using single-pixel detectors capable of operating at GHz-scale speeds. This spatial mask enables the network to leverage spatial information during the design and optimization processes. The modulation parameters are trained and optimized electronically, allowing precise manipulation of the light fields based on their spatial and temporal features. This configuration ensures that both spatial and temporal aspects of the light field are utilized, facilitating accurate and efficient pattern recognition through an all-optical framework. These advancements position the ST-AONN as a promising solution for multi-dimensional light field manipulation, signal processing, and pattern recognition in real-time and high-speed application scenarios.

## Results

### Principle and structure of ST-AONN

The specific structure of ST-AONN is shown in Fig. 1b. The system is formed by a 4*f* configuration consisting of grating-cylindrical lens pairs^41–45^. The light source adopts a Ti: Sapphire femtosecond (fs) laser with a bandwidth of ~6 nm and a pulse duration of ~200 fs. Firstly, a spatial mask is applied to the fs laser to generate the input pattern, with the input light field information in the spatial domain (x-y plane). The input light field is then spatially dispersed in frequency by grating 1 and collimated by cylindrical lens 1, thereby achieving a spatial Fourier transform. Therefore, the frequency-spatial frequency domain (*ω*-*k*_*y*_ plane) is formed at the rear focal plane (Fourier plane) of the cylindrical lens 1. Then, an ONN with diffraction layers is constructed in the *ω*-*k*_*y*_ domain. The spatially dispersed frequencies are recollected by cylindrical lens 2 and grating 2, and an inverse spatiotemporal Fourier transform is performed to transform the output light field into the spatiotemporal domain (*t*-*y* plane). Thus, the spatial input with chirp-free pulses is modulated into a spatiotemporal output with chirped pulses^45–47^. Unlike traditional diffractive neural networks operating in the spatial domain, this design allows light fields of different frequencies to be imparted with different diffraction phases, thereby achieving temporal chirp and enabling spatiotemporal modulation. The temporal chirp of the output pulse is determined by the diffraction layers in the system. Consequently, the output pulse chirp can be precisely designed by training the parameters of these diffraction layers. The optical forward propagation model of ST-AONN can be constructed based on this modulation process. See Supplementary Note 1 and Note 2 for more details about the light field analysis and forward propagation model of ST-AONN.

The spatiotemporal light field propagation of the ST-AONN system is shown in Fig. 1c. The inputs of ST-AONN are chirp-free pulse light fields with different spatial intensity distributions. After passing through ST-AONN, the input fields are modulated into spatiotemporal light fields with specific structures, which can be optimized through training according to the network target (the expected spatiotemporal light field structure output of the network). Then, the spatial mask on the *x*-*y* plane performs spatial filtering on the spatiotemporal structured light field, and the temporal intensity variation of the output field can be detected by a single-pixel PD. Therefore, the input spatial light field information is projected into the output temporal intensity variation information. Followed by simple digital processing, decisions can be made. Based on this, the network target can be designed according to the output pulse shape, such as the peak number of the pulse. Take handwritten digits as an example: when the input is “0”, the output pulse contains one peak; when the input is “1”, the output pulse contains two peaks, and so on.

### Spatiotemporal light field measurement

**Fig. 2:**
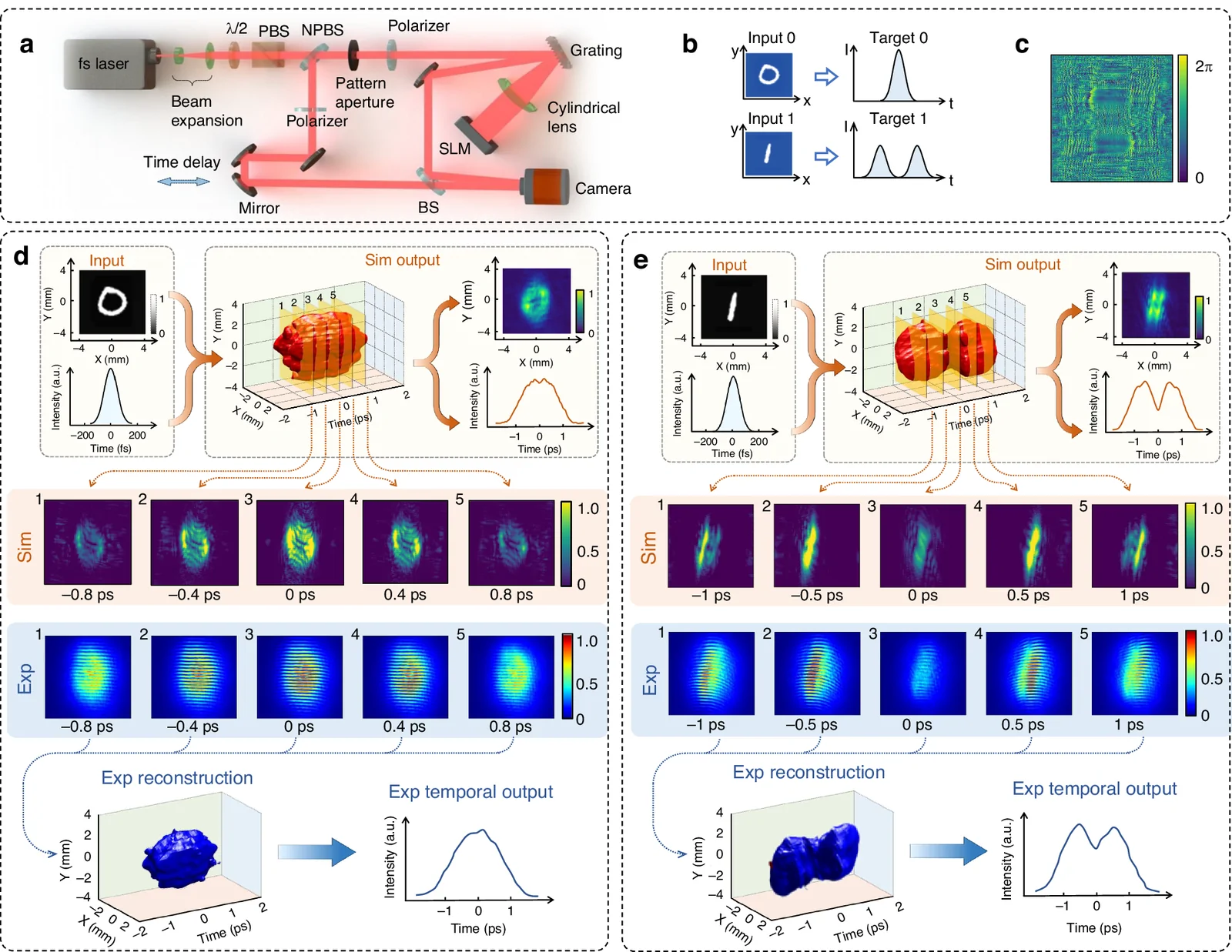
Experimental setup and results of spatiotemporal light field measurement. **a** Schematic diagram of the experimental setup. **b** Target design of the network, that is, different inputs correspond to different peak numbers of the output pulse. **c** The trained phase distribution loaded on the SLM. **d** The simulation and experimental results of the output field in the spatiotemporal domain when the input is a handwritten digit “0”. **e** The simulation and experimental results when the input is a handwritten digit “1”. The red profiles in (**d**) and (**e**) represent the 3D iso-intensity profile of the output light field. The maximum intensity is set to 1, and the isovalue is 0.03, which means that the isosurface profile includes all points with an intensity larger than 0.03. The space domain output distributions are the sum of the light field intensity in the time axis (the time axis range is −2 to 2 ps). The time-domain output pulse forms are the sum of the light field intensity in the *x*-*y* plane (the ranges of the *x*-axis and *y*-axis are both −4 mm to 4 mm; this square area performs spatial filtering on the spatiotemporal fields). The 1~5 simulation and experimental results correspond to the 1~5 temporal slices in the simulated output spatiotemporal field. λ/2 half-wave plate, PBS polarization beam splitter, NPBS non-polarization beam splitter

The feasibility of ST-AONN was analyzed through simulation and experiments. To detect the spatiotemporal structure of the light field, an interferometer is adopted^44,46^, the experimental setup is depicted in Fig. 2a. This interferometric system employs an unchirped reference pulse to conduct time-resolved, slice-by-slice measurements of the spatiotemporally modulated target field, enabling complete reconstruction of the spatiotemporal light field structure. The laser beam from the Ti: Sapphire fs laser is divided into two parts and directed to a modulation path and a reference path after the beam expansion and energy control, respectively. Inputs are loaded and switched through the 3D-printed pattern apertures. The modulation path contains a single-layer ST-AONN with a reflective spatial light modulator (SLM) loaded with a trained phase pattern that serves as the diffractive layer to generate target spatiotemporal structured light fields. For the reference path, the temporal width of the reference pulse is basically identical to the laser source, about 200 fs. After the time delay, the reference pulse overlaps with the chirped target field at an angle on the camera, forming interference fringes. The interference pattern is the spatial distribution of the chirped target field temporally sliced by the reference pulse. The temporal width of the chirped target light fields is set to approximately 2 ps, which is about 10 times that of the chirp-free reference pulse. This temporal width is suitable for slice detection in interferometric systems, while remaining compatible with the performance of the ST-AONN architecture. When the reference pulse scans over time, the distribution of the chirped target field in the spatiotemporal domain can be reconstructed. Refer to the “Materials and methods” section and Supplementary Note 3 for more details on the experimental setup and measurement principles.

The network first performs a binary classification task based on a “0/1” handwritten digit dataset, trained according to the target designed in Fig. 2b, where the temporal outputs are designed to have different peak numbers for different input digits. The trained phase distribution is shown in Fig. 2c. See the “Materials and methods” section and Supplementary Note 4 for the training method. Figure 2d and e show the simulation and experimental classification results of handwritten digits “0” and “1” using a single-layer ST-AONN. For the input beam, the temporal distributions are identical and consistent with the chirp-free fs laser, and the difference lies only in the spatial distribution, that is, the difference between handwritten digits “0” and “1”. The simulated 3D iso-intensity profiles show the spatiotemporal distributions of the output fields, and their spatial and temporal distributions can be calculated and shown in the upper-right part. The 1~5 simulation results correspond to the 1~5 temporal slices in the simulated output spatiotemporal field. By comparing the simulation outputs of “0” and “1”, it can be found that the output spatiotemporal structures are significantly different, and temporal distributions show clearly that their pulse forms have one and two peaks, respectively, which agrees well with the network target.

The lower parts of Fig. 2d, e show the experimental results. The 1~5 experimental measuring results also correspond to the 1~5 temporal slices. Comparison between the simulated and experimental temporal slice results shows good agreement in both intensity distribution and its temporal evolution across all slices. The interference fringes observed in the experimental data result from the interference between the target and reference light fields during measurement, which can be effectively removed through Fourier filtering (see Supplementary Note 3 for details). The experimental reconstruction results (blue profiles in Fig. 2d, e) were obtained by measuring the light field at 20 distinct temporal positions spaced at 0.2 ps intervals, followed by digital reconstruction. The experimental temporal output pulses are the sum of the reconstruction results in the x-y plane. Comparison shows that the experimental results agree well with the simulation results; the output pulse of input digit “1” has two peaks instead of one peak for input digit “0”. These results confirm that the network can effectively modulate the spatiotemporal distribution of the incident beam and transfer it to the required structure according to the design. In the following sections, it will be shown that together with spatial filtering and temporal intensity measurement, it is possible to perform computing tasks such as classification with this network.

### Handwritten digit classifier based on ST-AONN

**Fig. 3:**
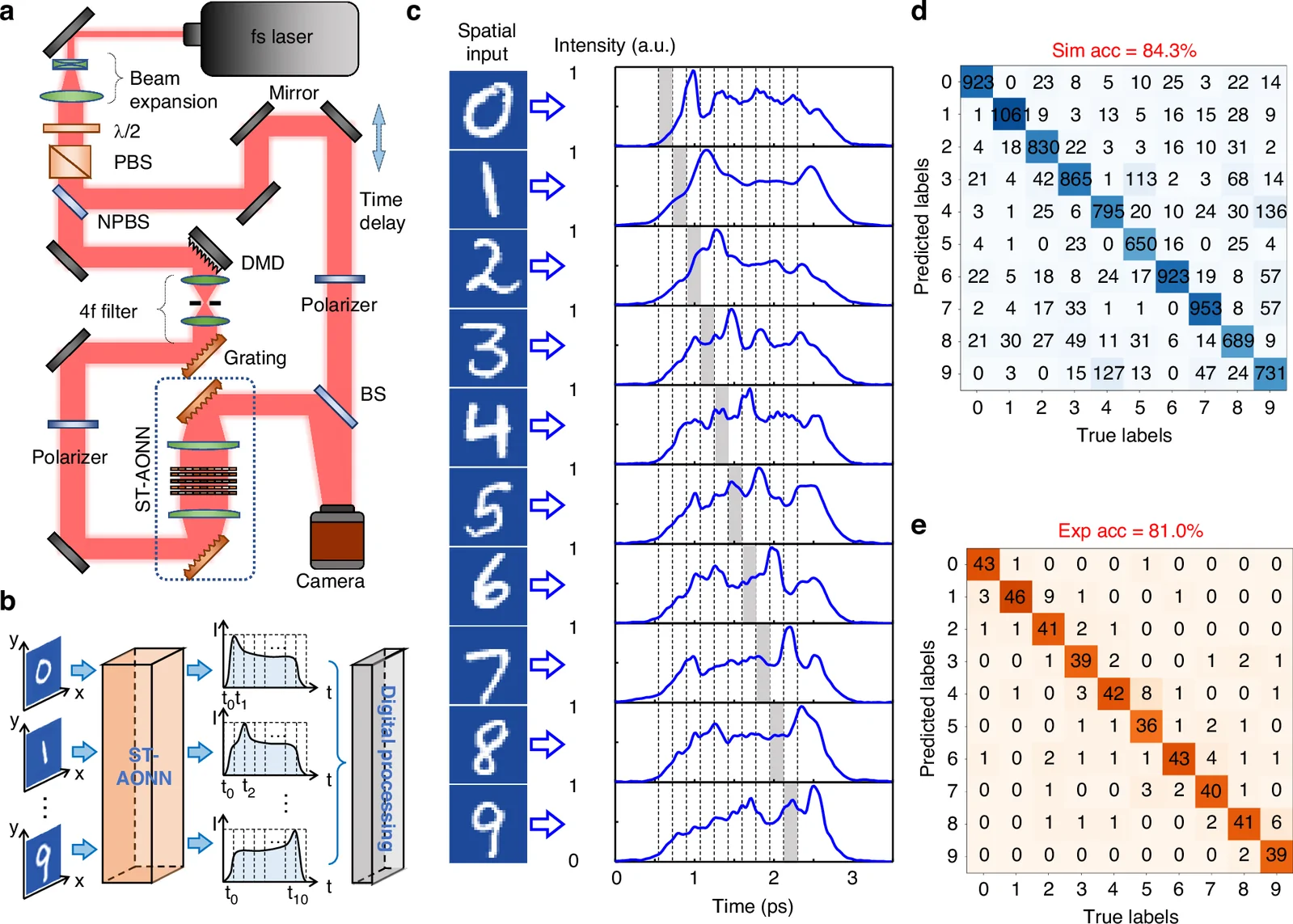
Classification studies of the MNIST handwritten digit database. **a** Schematic diagram of the ST-AONN system with 5 diffraction layers. **b** Schematic diagram of ST-AONN handwritten digit classifier based on pulse peak temporal position difference target design method. **c** 10 input digits in the MNIST test dataset with different labels and the corresponding output results in the time domain. **d** Confusion matrix of the simulated digit classifier. **e** Confusion matrix of the experimental digit classifier

Proof-of-concept studies on classification tasks were conducted based on the MNIST handwritten digit database. For 10-class classification, an ST-AONN system incorporating 5 diffraction layers was implemented, as shown in Fig. 3a. To facilitate the input of extensive pattern sets, a DMD was employed as the spatial input generator, followed by a 4f system and a grating to eliminate multi-level diffraction and compensate for the dispersion induced by the DMD. A 5-layer ST-AONN was constructed using transmissive liquid crystal diffraction optical elements (DOE) as the diffraction layers. To simplify the network target and reduce the computational demands on the network (discussed in Supplementary Note 5), the network is designed to generate output pulses with intensity peaks located at different temporal positions for different input digits, as shown in Fig. 3b. The temporal positions (*t*_1_~*t*_10_) of the output pulse intensity peaks correspond to different handwritten digits (0~9). This digit classifier consists of 5 diffraction layers, each of which is a phase-only transmission diffractive layer. The input field is a pulse with a linear chirp in the time domain (full width at half maximum (FWHM) ~1.3 ps). The maximum intensity in 10 temporal regions is selected as the classification criterion. The loss function in training adopts the cross-entropy (CE) method^48–51^, which can be expressed as:

1 $$Los{s}_{CE}=-{\sum }_{i=1}^{n}{y}_{i}\log {\hat{y}}_{i}$$

where ${y}_{i}$ and ${\hat{y}}_{i}$ represent the probability distribution of the target and the network output, respectively, and *n* is the number of categories. A circular hole mask is set in the output spatial domain for spatial filtering, and the diameter of the circular hole is the side length of the input image. With this design, ideally, the change of the spatiotemporal light field in the network is shown in Supplementary Fig. 4 in the Supplementary Information. The network will gradually concentrate the light energy at different temporal positions for different input digits.

After training, the design of the digit classifier was numerically tested using 10,000 images from the MNIST test dataset. Figure 3c shows 10 spatial inputs (with labels 0~9) from the MNIST test dataset and the corresponding output results in the time domain. During testing, digital processing (including temporal integration and value comparison) is applied to the temporal outputs to determine the final classification decision. Figure 3d shows the simulation confusion matrix of the digit classifier, with an accuracy of 84.3%. In addition, we conducted experimental testing using an interferometric system on a handwritten digit dataset, achieving an accuracy of 81.0%, as shown in Fig. 3e. Although ST-AONN’s recognition accuracy currently trails that of traditional diffractive neural networks (see Supplementary Note 6 for detailed results), this limitation arises from its capability to process spatiotemporal information simultaneously during diffraction. Nevertheless, several strategies can effectively enhance its accuracy, including target optimization, unsupervised learning methods, and increasing the network scale. As described in Supplementary Note 7, these methods have achieved significant results, increasing the recognition accuracy of handwritten digit datasets to over 90%. In addition, the evolution of the spatiotemporal light field structure and temporal pulse waveform in ST-AONN through 5 diffraction layers can be found in Supplementary Note 8. Moreover, the classification study was also conducted on the MNIST fashion database, and the recognition accuracy reached 71.6%, see Supplementary Note 9 for details.

### Spatiotemporal optical signal processing with high-speed ST-AONN

In this section, the application of ST-AONN to lidar systems is presented, showcasing its effectiveness in accelerating spatiotemporal optical signal processing for practical implementations. The operational principle of lidar involves emitting a probe light toward the target and detecting the reflected signal using a single-pixel or an arrayed detector. The detected signal typically has both space and time information (spatiotemporal signal). The spatial position of the target is determined from the location where the reflected pulse is received, while the distance to the target is calculated based on when the pulse is received (time-of-flight). However, most lidar systems rely on scanning mechanisms (like MEMS) to achieve detection in 2D or 3D scenes, which increases system complexity and limits its high-speed performance^52–54^. In contrast, the ST-AONN lidar enables scanning-free multi-target detection and scene reconstruction, offering significant advantages in both system simplicity and high-speed performance.

**Fig. 4:**
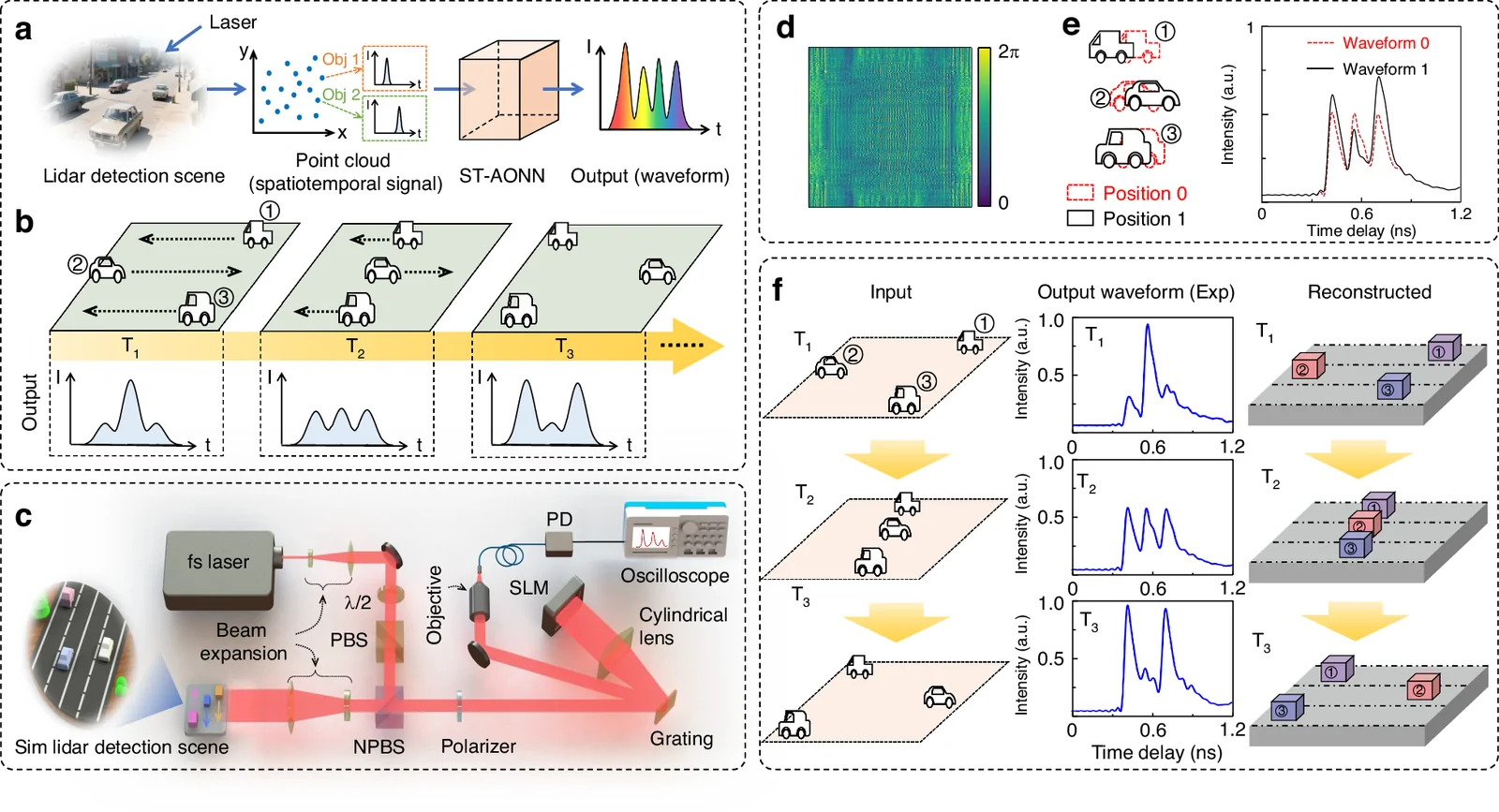
Lidar application based on ST-AONN. **a** Schematic diagram of the ST-AONN lidar system. **b** Schematic diagram of the real-time motion monitoring based on ST-AONN lidar. **c** Schematic diagram of ST-AONN lidar setup. **d** The phase distribution loaded on the SLM. **e** Relationship between target positions and output waveform. Multiple targets move from position 0 to position 1, and the corresponding output pulse changes from waveform 0 to waveform 1. **f** Target position recovery results at different times based on experimental output pulses

The schematic illustration of the system is shown in Fig. 4a. The laser illuminates the target scene, and the reflected signal (the so-called point cloud) containing space and time features is processed by ST-AONN. After propagating through the ST-AONN, the three-dimensional position information of the target is projected into a temporal pulse intensity waveform. By analyzing this waveform, the positions of the target cars in the detected scenario can be extracted. This information, provided by the lidar system, can be utilized to support real-time decision-making in applications such as autonomous driving. More specifically, the waveform of the output pulse changes with the displacement of detected targets, thereby realizing real-time motion monitoring, as illustrated in Fig. 4b.

Figure 4c is the schematic illustration of the ST-AONN lidar setup. The laser beam undergoes a two-stage expansion, from the original 1 mm diameter to 10 cm, to achieve sufficient scene illumination. The expanded beam then illuminates the detected scene (positioned ~50 cm away), and the reflected signals are collected by the same lens before entering the signal processing system. We implemented three movable reflectors (8 mm × 5 mm each) to simulate moving vehicles, with a travel range of approximately 8 cm and speeds ranging from 10 to 50 mm/s. A single-pixel PD and a high-speed oscilloscope are used to detect the temporal output signals, and a dataset was constructed to emulate the scene of multiple vehicles driving on the road and to train the network. See the “Materials and methods” section for more details. Figure 4d is the phase distribution obtained from training. Figure 4e illustrates the relationship between target positions and the output waveform. As can be observed, when targets move, the waveform changes, and object position detection can later be achieved by analyzing the waveform. In this case, the refresh speed of the output is determined by the bandwidth of the PD, which is 30 GHz in the experiment.

Figure 4f demonstrates the reconstructed target position with the ST-AONN. Comparison with the input ground truth shows a positional deviation within 2 mm and a velocity discrepancy below 1.5 mm/s between reconstructed and actual scenes. The positioning error is less than 2.5%, and the speed error is less than 3.0%. For detailed digital processing and scene reconstruction procedures, please refer to Supplementary Note 10. For enhanced visualization, a supplementary movie (Supplementary Movie 1 in Supplementary Information) that synchronously displays the actual lidar detection scene, output pulse waveform, and real-time recovered results is also provided, which effectively demonstrates the high-speed signal processing and real-time recovery capabilities of the ST-AONN lidar.

Nevertheless, we have also demonstrated theoretically that the proposed system has potential in orbital angular momentum (OAM) mode demultiplexing, based on the high-speed OAM pulse laser source^55,56^. It is demonstrated that the operation speed of the ST-AONN demultiplexer can reach the GHz level, see Supplementary Note 11 for details. Furthermore, we experimentally validated the high-speed ST-AONN system with a DMD as the input device for aircraft takeoff and landing detection. A specialized dataset simulating aircraft altitude and attitude variations during takeoff and landing phases was constructed, and motion detection tasks were performed based on this dataset. Detailed descriptions of the experimental setup, dataset characteristics, and results are provided in Supplementary Notes 12–15.

## Discussion

Benefiting from the inherent light-speed processing capability of diffractive neural networks, the actual operational speed is ultimately limited by the performance of the signal input and detection. The ST-AONN system employing temporal detection achieves significant improvement in processing throughput, with theoretical operational speeds reaching the GHz level. In practical lidar applications, which do not require external signal input devices such as DMDs, the system speed is constrained by the laser repetition rate (76 MHz). Nevertheless, this configuration still delivers a substantial performance gain over traditional diffractive neural networks that rely on 2D array detectors, which typically operate in the 10²~10³ Hz. In addition, the ST-AONN lidar performs most of the computational operations as light propagates through the diffractive network, substantially reducing the digital processing requirements compared to traditional lidar systems. In terms of computational performance, the ST-AONN lidar delivers a processing capacity of 778.3 TOPS (tera-operations per second)^20,57–59^ with a theoretical computational energy efficiency exceeding 10³ TOPS/W (tera-operations per second per watt); refer to Supplementary Note 16 for further analysis. Overall, the ST-AONN lidar system demonstrates significant advantages over conventional lidar architectures by increasing system bandwidth while reducing energy consumption. Besides, the proposed system has reduced the need for a fast scanning system, which would also be beneficial for practical implementations. However, the scanning-free architecture also has some drawbacks, such as the need for high-energy pulsed laser sources, reduced irradiance per unit area when illuminating large scenes, and the need for higher-performance and larger-scale diffraction neural networks to handle complex detection scenes. A comprehensive analysis of system operational speed and energy efficiency is provided in the “Materials and methods” section.

In conclusion, we presented a novel ST-AONN system that incorporates the time dimension into the traditional space-only diffractive neural networks, thus enabling spatiotemporal multi-dimensional light field modulation and signal processing. Theoretical and experimental results are presented to demonstrate that ST-AONN can generate specific spatiotemporal structured output fields according to the input spatial and temporal features. The training and testing results based on the MNIST datasets demonstrate that ST-AONN exhibits good performance, suggesting that the classification performance of the network will likely improve with the addition of more network layers. The real-time signal processing performance of the system in the lidar application is also verified. Experimental results show that ST-AONN enables multiple targets’ motion monitoring by tracking the output waveform variations and further achieving real-time scene reconstruction.

This innovative approach not only provides enhanced processing capabilities but also overcomes the bandwidth limitations of traditional systems, laying the foundation for the future development of multi-dimensional, highly flexible, and high-speed AONN. Compared to existing space-to-time information projection approaches, the ST-AONN system actively modulates both spatial and temporal dimensions, enabling simultaneous encoding and processing of the spatiotemporal information of the light field. Additionally, since the proposed scheme requires only a single photodiode instead of an array imaging sensor, the system can be easily adapted to other electromagnetic bands beyond visible light. For instance, the ST-AONN system based on broadband light sources in the infrared, terahertz, microwave, and other bands can eliminate the need for the complex and costly preparation of large-area 2D imaging devices in these bands. Furthermore, this architecture offers several promising methods for introducing nonlinearity to enhance its processing capabilities. It is known that high peak power (short pulse laser) is necessary for exciting optical nonlinearity, and ST-AONN using a fs laser as the source is clearly beneficial for this aspect. Moreover, the modulation of the system can arbitrarily control the pulse shape of the light field, thereby providing a controllable approach to introduce nonlinearity in the network^60–62^. From an integration perspective, with the development of integrated photonic technologies, such as on-chip dispersion, collimation, waveguides, and manipulation devices, the realization of an on-chip ST-AONN system appears highly promising^63–65^. In short, these developments establish the ST-AONN as a transformative platform for multi-dimensional optical modulation, signal processing, and pattern recognition in real-time and high-speed application scenarios.

## Materials and methods

### The forward propagation model of ST-AONN

The forward propagation model of ST-AONN is constructed based on the 4*f* configuration consisting of grating-cylindrical lens pairs and implemented with TensorFlow. The fs laser used in the experiment has a pulse width greater than 100 fs, so the propagation of the laser in low-dispersion media such as air and lenses can be considered to be chirp-free. The chirp in the system is only generated by the grating and the diffraction layers, where the grating spatially disperses the frequencies and the diffraction layers generate chirping in the frequency-spatial frequency domain. The connection between gratings, lenses, and diffraction layers is achieved through free-space diffraction. For detailed theoretical analysis, please refer to the Supplementary Note 2.

### Training of ST-AONN

The ST-AONN was trained using Python version 3.6.5 and TensorFlow framework version 2.4.0 (Google Inc.) on a server (NVIDIA GeForce RTX 3090 GPU and Intel(R) Xeon(R) Silver 4210R CPU @2.40 GHz with 32 GB RAM, running a Windows 10 operating system). In training, to balance the computational accuracy and the computing power, the light field is set to a 200 × 200 × 200 matrix, corresponding to the *x*-axis, *y*-axis, and *ω*-axis, respectively. The phase distributions of the diffraction layers are the optimization target, and the results are 200 × 200 matrices (0~2π), corresponding to the spatial domain of the light field. The loss function was constructed with the CE or MSE method, and the optimizer uses the Adam optimizer. See Supplementary Note 4 for more details.

### Experimental setup of spatiotemporal structured light field measurement

The laser source used in the experiment is an ultrafast Ti: Sapphire laser (COHERENT Mira 900-D, which operates at a wavelength of 800 nm with a bandwidth of ~6 nm, FWHM ~ 200 fs in the experiment, repetition rate ~76 MHz, ~1 W) with linearly polarized output. The laser spot diameter is expanded from ~1 mm to ~20 mm by a concave and convex lens pair, and then an aperture is used to select the center part to ensure that the light field is as close to a plane wave as possible. A rotatable half-wave plate and a PBS control the laser energy. Then the laser beam is divided into a modulation path and a reference path by an NPBS.

In the modulation path, the laser passes through the pattern aperture to generate the specific spatial distribution and then enters the single-layer ST-AONN. The patterned aperture is prepared by high-precision 3D printing and manually switched. The grating is a 1200 line/mm blazed grating, and the focal length of the cylindrical lens is 500 mm. A reflective SLM (Meadowlark Optics E19×12, 1920 × 1200 resolution with 8 μm pixel size, optical energy utilization exceeds 95%) is used as a single diffraction layer, which is a phase-only modulation device composed of liquid crystal cells. Since the phase distribution obtained by training is a 200 × 200 matrix, we load the adjacent 5 × 5 matrix on the SLM with the same phase to form a 1000 × 1000 matrix phase distribution, which can expand the physical spatial size of the light field. In the reference path, the time delay in the reference path is achieved by two mirrors on a displacement stage (Thorlabs, LTS300C, 300 mm travel range, 0.1 μm minimum incremental movement). The two mirrors are at an angle of 90°, and the displacement accuracy of the displacement stage is within the μm range to achieve fs-scale time delay accuracy. Polarizers in two paths are used to adjust the polarization of the light fields. The camera is a CMOS monochrome camera (Thorlabs CS505MU, 2448 × 2048 resolution), and image acquisition is achieved through the software on the PC.

### Experimental setup of ST-AONN lidar

The laser used in the ST-AONN lidar is the same as that used in the spatiotemporal light field measurement system. After beam expansion and energy control, the laser is irradiated onto the simulated scene of roadway vehicle motion, that is, multiple movable reflectors (8 × 5 mm mirrors are fixed on multiple displacement stages, OptoSigma, OSMS(RE), 50 mm travel). After coaxial beam contraction, the reflected light enters the subsequent signal processing module, as shown in Fig. 4C. The input light fields pass through the single-layer ST-AONN (the models and parameters of the devices in the network are consistent with those in the spatiotemporal light field measurement system), and the output field is coupled into an optical fiber (multimode, 1 m long) by an objective lens (Olympus, 20×, NA = 0.25). Then high-speed detection is performed using a single-pixel PD (Thorlabs DXM30BF, 30 GHz) and an oscilloscope (Keysight DSAV164A, 16 GHz analog bandwidth, 80 GSa/s sample rate). The spatial filtering of the output spatiotemporal field in this system is achieved through the aperture of the objective lens.

### System operating speed and energy efficiency analysis

In the interferometric ST-AONN, the operation speed is relatively low due to the need for spatiotemporal slicing and subsequent imaging detection. In contrast, the high-speed ST-AONN system employing single-pixel PD measurement benefits from light-speed signal processing within the diffractive network. Consequently, its overall speed is constrained by signal input and detection capabilities, specifically the laser repetition rate, the switching speed of the input device (e.g., DMD), and the bandwidth of both the PD and oscilloscope. In the experiment, the bandwidth of the PD and oscilloscope (GHz level) substantially exceeds the laser repetition rate (MHz level) and DMD switching speed (kHz level), so the calculation speed is usually limited by the encoding speed of the inputs. In practical ST-AONN lidar scenarios for real-world scene detection, the system speed would instead be limited by the laser repetition rate of 76 MHz.

Taking the single-layer ST-AONN lidar configuration with 200 × 200 modulation units in Fig. 4c as an example, the system achieves a frame rate of 76 Mfps, performing 3.04 trillion operations per second in its forward model. For image processing, this corresponds to a computational throughput of 778.3 TOPS. When extended to a 5-layer architecture, the system’s processing capacity increases to 3891.2 TOPS. Experimentally, with a laser input power of 1 W, the theoretical computational energy efficiency exceeded 10³ TOPS/W. When accounting for the system power consumption—including the laser source (~400 W), SLM (~20 W), single-pixel PD (~1 W), totaling approximately 500 W—the overall system energy efficiency reaches about 7.8 TOPS/W.

### Vehicle and aircraft motion datasets

The vehicle and aircraft motion datasets simulate the motion of vehicles and aircraft, respectively. The vehicle motion dataset is constructed by changing the position of 20 vehicle profiles, containing 2500 samples (2000 for training and 500 for testing). Each sample contains three types of vehicles in the upper, middle, and lower positions, 200 × 200 pixels, the length of the vehicle is ~60 pixels, and the height is ~40 pixels. The position has a random difference of ±10 pixels in the vertical and horizontal direction. The aircraft motion dataset is constructed by changing the altitude and pitch angle of 20 aircraft profiles, containing 2500 samples (2000 for training and 500 for testing) located at five different altitudes, with corresponding pitch angles are 0°, 15°, 30°, 15°, and 0°. Each sample contains 200 × 200 pixels, the length of the aircraft is ~100 pixels, and the height is ~50 pixels. The aircraft position has a random difference of ±10 pixels in the vertical direction and ±50 pixels in the horizontal direction. The aircraft pitch angle also has random differences of ±10°.

## Supplementary information

supplementary information

Movie 1

## Data Availability

All data needed to evaluate the conclusions in the paper are available in the manuscript or the Supplementary Information. The custom dataset used for network training is publicly available in the Zenodo database under the link: https://doi.org/10.5281/zenodo.17809138.

## Code availability

The source code is publicly available in the Zenodo database under the link: https://doi.org/10.5281/zenodo.17809138.
