# Supplementary material for: Spatiotemporal all-optical diffractive neural networks empowered by spatiotemporal light field manipulation
Source: Light Sci Appl. 2026 Sep 16;15:382. doi: 10.1038/s41377-026-02366-7 (PMC13578322; doi:10.1038/s41377-026-02366-7)
Supplement: Supplementary file 1 [file 41377_2026_2366_MOESM1_ESM.docx]

Supplementary Information for

Spatiotemporal all-optical diffractive neural networks empowered by spatiotemporal light field manipulation

Fu Feng^1,2,^†, Ziyang Zhang^1,^†, Dewang Huo^3^, Xiaolong Li^1^, Qinggang Lin^4^, Xingyu Zhao^2^, Guozhong Hou^1^, Daoxin Dai^2^, Michael G Somekh^1,5,^* Xiaocong Yuan^1,6,^*

^1^Research Center for Frontier Fundamental Studies, Zhejiang Lab, Hangzhou 311100, China

^2^State Key Laboratory of Extreme Photonics and Instrumentation, College of Optical Science and Engineering, Zhejiang University, Hangzhou 310027, China

^3^Zhejiang Key Laboratory of 3D Micro/Nano Fabrication and Characterization, Westlake Institute for Optoelectronics, Hangzhou, Zhejiang 311421, China

^4^College of Optical and Electronic Technology, China Jiliang University, Hangzhou, Zhejiang 310018, China

^5^Faculty of Engineering, University of Nottingham, Nottingham NG7 2RD, U.K.

^6^Key Laboratory of Optoelectronic Devices and Systems of Ministry of Education and Guangdong Province, College of Physics and Optoelectronic Engineering, Shenzhen University, 518060 Shenzhen, China

† These authors contributed equally to this work.

* Corresponding author. Email: Michael.Somekh@nottingham.ac.uk (M. G. S); xcyuan@szu.edu.cn (X. Y)

**Contents:**

**Supplementary Notes:**

1. Light field analysis in ST-AONN
2. The forward propagation model of ST-AONN
3. Principle of spatiotemporal light field measurement
4. Training of ST-AONN
5. Target design based on the number of peaks
6. Comparison of ST-AONN and diffraction AONN Performance
7. Performance improvement analysis of ST-AONN
8. Spatiotemporal light field structure evolution in ST-AONN with 5 diffraction layers
9. Classification study based on the MNIST fashion dataset
10. The digital processing and scene reconstruction of the ST-AONN lidar
11. ST-AONN OAM demultiplexer for high-speed optical communication applications
12. Implementation of high-speed ST-AONN
13. High-speed ST-AONN
14. Performance differences between high-speed ST-AONN and interferometer-based ST-AONN
15. Effect of spatial asymmetry on ST-AONN
16. System energy efficiency analysis

**Supplementary Figures:**

Fig. S1. Schematic of the ST-AONN

Fig. S2. Schematic diagram of measuring the target spatiotemporal field

Fig. S3. Matrix dimensions in ST-AONN numerical training

Fig. S4. Simulation results of the target design based on the number of peaks

Fig. S5. The simulation results of a space-only diffraction AONN

Fig. S6. Improving the performance of ST-AONN by appropriate target design

Fig. S7. Unsupervised learning method based on clustering training

Fig. S8. Schematic diagram of spatiotemporal light field changing in 5-layer ST-AONN

Fig. S9. Classification studies of MNIST fashion database

Fig. S10. The digital processing and scene reconstruction of the ST-AONN lidar

Fig. S11. ST-AONN OAM demultiplexer

Fig. S12. High-speed ST-AONN

Fig. S13. The structure and switching of DMD

Fig. S14. Classification studies of aircraft motion dataset based on the interferometer-based ST-AONN

Fig. S15. Effect of spatial asymmetry on the performance of ST-AONN

Table S1. Energy efficiency comparison of partial different optical computing architecture and electronic hardware

**Supplementary Movie:**

Legends for Supplementary Movie 1

**Other Supplementary Information includes the following:**

Supplementary Movie 1

Supplementary Note 1. Light field analysis in ST-AONN

The structure of ST-AONN is a 4*f* system consisting of grating-cylindrical lens pairs. As shown in Fig. 1B in the manuscript, the spatial intensity of the input light field is distributed in the x-y plane, and the light field propagates along the z-axis. In addition, the input light field is also distributed in the frequency domain (*ω*) and the time domain (*t*), and the two are time-frequency Fourier transform relationships. The input light field passes through grating 1, and its frequency component of the incident light field is expanded along the *x*-axis, and then focused by cylindrical lens 1 to form a frequency-spatial frequency domain (*ω*-*k_y_* plane) on the focal plane. Grating 1 and cylindrical lens 1 perform spatiotemporal Fourier transform on the light field. It should be noted that the spatial Fourier transform is not achieved through lenses but through free-space propagation in the system. The spatially dispersed frequencies are collected again by cylindrical lens 2 and grating 2, and the inverse spatiotemporal Fourier transform is performed to transform the output light field into the spatiotemporal domain (*t*-*y* plane)^1,2^.

The diffraction neural networks in ST-AONN is similar to the spatial-only diffraction neural networks, each diffraction unit on the diffraction layers can be regarded as a neuron in the network, and the neurons are interconnected through optical diffraction. The complex transmission/reflection coefficients of the diffraction units are learnable network parameters, which are iteratively adjusted during the training process. However, unlike spatial-only diffraction neural networks whose diffraction layers are built in the spatial domain (*x*-*y* plane), the diffraction layers in ST-AONN are built in the frequency-spatial frequency domain (*ω*-*k_y_* plane). Based on this, ST-AONN can simultaneously modulate the spatial and frequency domain components of the light field, thereby further realizing spatiotemporal light field modulation.

Supplementary Note 2. The forward propagation model of ST-AONN

ST-AONN consists of grating-cylindrical lens pairs and diffraction layers, as shown in Fig. S1. The spatiotemporal profiles in the ST-AONN are theoretically analyzed step by step^3-7^. For simplicity, a Fourier transform limited pulse with Gaussian spatial profile and Gaussian spectral distribution is chosen as the input ultrafast field, and the wave packet can be expressed as

$E_{1}(x,y,\omega)=exp\left(-\frac{x^{2}+y^{2}}{a^{2}}\right)exp\left(-\frac{\left(\omega-\omega_{c}\right)^{2}}{b^{2}}\right)$ (S1)

where $\omega_{c}$ is the optical carrier frequency, $\omega_{c}=2\pi c/\lambda_{c}$, *c* is the light speed in vacuum, *λ_c_* is the central wavelength, *a* and *b* are the beam waist radii of the Gaussian profile in the spatial domain and frequency domain, respectively. The input light field passes through an image mask to generate a specific spatial light field distribution, which can be represented as

$E_{2}(x,y,\omega)=E_{1}(x,y,\omega)T_{input}\left(x,y\right)$ (S2)

where *E*_2_ is the field immediately after the mask, and $T_{input}$ is the transmission function of the image mask, i.e., the spatial information of the input image. Based on the angular spectrum propagation diffraction theory, the propagation of light field in free space can be expressed as

$E_{3}(x,y,\omega)=\Im^{-1}\left\{\Im\left\{E_{2}(x,y,\omega)\right\}H\left\{f_{x},f_{y},\omega\right\}\right\}$ (S3)

where the transfer function $H\left\{f_{x},f_{y},\omega\right\}=exp\left(ikz-i\pi\lambda z\left(f_{x}^{2}+f_{y}^{2}\right)\right)$ is the transfer function, $k=2\pi/\lambda$ is the wave vector, $z$ is the propagation distance of the light field. $\Im$ and $\Im^{-1}$ denote the spatial Fourier transform and inverse Fourier transform. *f_x_*, *f_y_* are spatial frequencies in *x* and *y* directions, respectively. For ultrafast laser with pulse widths greater than 100 fs, the effect of air dispersion can be ignored in short-distance propagation, and the laser propagation in air can be considered chirp-free. Therefore, $\lambda$ in the transfer function can be considered equal to *λ_c_*, and the transfer function is independent of *ω*. All light fields propagating in free space in the system can be calculated according to Eq. S3.

The diffracted input field *E*_3_ passes through the grating 1, the first order field immediately after the grating can be written as

$E_{4}(x,y,\omega)=E_{3}(\beta x,y,\omega)exp\left(i\gamma\left(\omega-\omega_{c}\right)x\right)$ (S4)

where $\beta=\cos\left(\theta_{in}\right)/\cos\left(\theta_{out}\right)$, $\gamma=2\pi/\omega_{c}d\cos\left(\theta_{out}\right)$, *θ_in_* and *θ_out_* are the incident angle and diffracted angle of the central wavelength ray with respect to the grating normal, respectively. $d$ is the periodicity of the grating. Eq. S4 indicates that the input field is dispersed frequencies spatially along the x-axis, and the grating line direction along the y-axis. The free space propagation from the grating 1 to the cylindrical lens 1 can be calculated according to Eq. S3, with the propagation distance of the focal length of the cylindrical lens *f*, and *E*_5_ at the front plane of cylindrical lens 1 is obtained. The field after cylindrical lens can be written as

$E_{6}(x,y,\omega)=E_{5}(x,y,\omega)exp\left(-\frac{ikx^{2}}{2f}\right)$ (S5)

then *E*_6_ continues to propagate in free space and reaches the position of the diffraction layers, the field before the diffraction layers is *E*_7_. For the first diffraction layer, its field modulation can be expressed as

$E_{8}(x,y,\omega)=E_{7}(x,y,\omega)T_{diff}\left(x,y\right)$ (S6)

where *T_diff_* is the transmittance function of the diffraction layer,, $A\left(x,y\right)$ and are the modulation functions of the diffraction layer on the field amplitude and phase in the x-y plane, respectively. For a phase-only modulation, the amplitude is assumed to be a constant. Then the light field propagates through free space and reaches the next diffraction layer. Therefore, the modulation of each diffraction layer can be described with Eq. S6 and Eq. S3. Furthermore, Eq. S6 also applies to the case of reflective diffraction layers, *T_diff_* becomes *R_diff_*, and also includes amplitude and phase modulation parameters.

The propagation of the light field after passing through the diffraction layers is consistent with the above, and the field sequentially passes through the cylindrical lens 2 and grating 2 to obtain the final output field $E_{out}$. The calculation can refer to Eq. S5 and Eq. S4 respectively. The $E_{out}$ can be detected using a high-speed single pixel PD for time domain signal detection, and the field in the time domain is the inverse Fourier transform of the field in the spectrum domain, then the intensity in the time domain can be calculated as

$I\left(x,y,t\right)=\left|\Im^{-1}\left\{E_{out}\left(x,y,\omega\right)T_{mask}\left(x,y\right)\right\}\right|^{2}$ (S7)

$I(t)=\iint I\left(x,y,t\right)dxdy$ (S8)

where the *T_mask_* is the transmission function of output mask, representing the spatial filtering of the output light field, as shown in Fig. 1C. Then, by integrating *x* and *y*, the optical intensity output results in time domain can be obtained. The above theoretical analysis describes the propagation of the light field in ST-AONN, which is the forward propagation model.

Supplementary Note 3. Principle of spatiotemporal light field measurement

The interferometric system, as shown in Fig. 2A, for spatiotemporal light field characterization employs an unmodulated reference pulse to conduct time-resolved, slice-by-slice measurements of the spatiotemporally modulated target field, thereby enabling complete reconstruction of the measured spatiotemporal field structure. After overlapping interference, the reference pulse and the target field overlap at an angle on the camera, and the two fields interfere in the overlapping area to form interference fringes^3,8^, as shown in Fig. S2. The direction and density of the interference fringes are determined by the angle direction and angle size of the target light field and the reference pulse, respectively. While, the spatial range and contrast of the interference fringes represent the overlapping range and relative intensity of the two fields, respectively. Usually, the chirped target field is longer than the chirp-free reference pulse in the time domain, so the temporal width of the overlap range is determined by the reference pulse. Therefore, if the spatial distribution of the reference pulse is set to a uniform field greater than the target field, the spatial range and contrast of the interference fringes can represent the spatial range and intensity distribution of the target field at the temporal overlapping position. By changing the temporal interference position of the reference pulse and the target field, that is, changing the time delay of the reference pulse in the reference path, the complete field distribution of the target light field in the entire spatiotemporal domain can be obtained. During post-processing of the experimental data, Fourier filtering can be applied to eliminate interference fringes from the measured results. The specific procedure is as follows: first, a 2D Fourier transform is applied to the image to obtain its frequency-domain distribution, which is typically displayed with the zero-frequency component shifted to the center. A 2D frequency-domain filter (such as a band-stop or Gaussian band-stop filter) is then applied to retain the zero-order spectral component. Finally, an inverse Fourier transform is performed on the filtered spectrum to reconstruct the fringe-corrected image.

Supplementary Note 4. Training of ST-AONN

Based on the forward propagation model of ST-AONN, the mapping relationship between the input and output of ST-AONN can be simulated. Compare the output of the network with the target (for which the network is being trained), and backpropagate the generated errors to iteratively update the diffraction layers. During training, a loss function was defined to evaluate the performance of the ST-AONN output with respect to the expected target, and the neural network parameters were iteratively optimized using the algorithm to minimize the loss function.

The loss function adopts the cross entropy (CE) or mean square error (MSE) method according to the target design, and the optimizer uses the Adam optimizer. Adam (Adaptive Moment Estimation) optimizer, which is a gradient-based optimization algorithm that can automatically update the learning rate based on the historical gradient information and the average value of each parameter^9^. It combines the stochastic gradient descent algorithm (SGD) and the adaptive learning rate algorithm to quickly converge and reduce training time.

In training, the input field $E_{1}(x,y,\omega)$ is set to a 200×200×200 matrix, with three dimensions representing the *x*-axis, *y*-axis, and *ω*-axis of the light field, respectively. The input image mask$T_{input}\left(x,y\right)$ and output mask $T_{mask}\left(x,y\right)$ are set to 200×200 matrixes to match the input light field, as shown in Fig. S3. The output field $I\left(x,y,t\right)$ is also a 200×200×200 matrix, with three dimensions representing the *x*-axis, *y*-axis, and *t*-axis of the light field, respectively. Training with this setting yields a diffraction coefficient of n×200×200 matrix, where n is the number of diffraction layers, and the range of the matrix elements is 0 to 2π. The training used a fixed learning rate = 0.02, batch size = 10, and epochs = 50. For multi-layer diffraction layer networks, the distance between diffraction layers can also be optimized as one of the parameters of the network. Since the diffraction layer phase distribution obtained through network training was physically produced by SLM, the physical size of the matrix elements was the real pixel size of the SLM.

Supplementary Note 5. Target design based on the number of peaks

For low-class classification tasks, both peak number and peak location decoding methods demonstrate comparable effectiveness, achieving similarly high recognition accuracy. However, as the number of classes increases, the peak number decoding method becomes progressively less suitable, exhibiting a significant decline in accuracy compared to peak location methods. As shown in Fig. S4, the five-layer ST-AONN designed with a peak number target achieves only 50.4% accuracy on the handwritten digit dataset. Moreover, recognition reliability decreases progressively as the peak number increases, complicating final classification decision-making.

This performance difference stems from the fundamental difference in their decoding mechanisms: peak location only requires the strongest feature peak in each category, whereas peak number requires an increasing number of peaks for each category, ranging from one peak for the digit “0” to ten peaks for the digit “9”. This escalation complicates the construction of the loss function and the differentiation of the output waveform, while also placing higher demands on the network capability. Therefore, under the same network architecture, the peak location decoding method achieves significantly higher accuracy than peak number decoding.

Supplementary Note 6. Comparison of ST-AONN and diffraction AONN Performance

Fig. S5 shows the simulation results of a space-only diffraction AONN without spatiotemporal modulation with same conditions (5 diffraction layers, 200×200 diffraction pixels, 50 mm diffraction layer spacing), the target design is shown in Fig. S5A. It can be seen that the recognition accuracy of space-only AONN on the MNIST handwritten dataset is 91.1%, while the accuracy of ST-AONN is 84.3% (as shown in Fig.3C in the manuscript). This is due to the fact that ST-AONN processes the spatial and temporal information simultaneously during the diffraction while diffractive AONNs process spatial features only, and it can modulate the spatial information of the input field more efficiently and thus achieve better accuracy.

Supplementary Note 7. Performance improvement analysis of ST-AONN

The recognition accuracy of ST-AONN can be improved by serval different approaches, such as 1) choosing more appropriate targets, 2) using an unsupervised learning method for target design, and 3) increasing the scale of the network.

1)	Choosing more appropriate target

In the manuscript, the network's target outputs follow a logical sequence to facilitate the demonstration. In the manuscript, for inputs with labels ranging from 0 to 9, their output targets were designed so that the peak position gradually shifts from left to right on the time axis. However, this design method is not optimized to align with the features of the inputs (e.g., digits ‘1’ and ‘7’ may share similar features but are forced to be separated in output targets). By adjusting the target, we can better align the output target with the input data's features, making it easier for the network to converge and reducing the demand on the network’s capability of modulating light.

To achieve better target, contrastive learning is employed to optimize the target design. This method can obtain the optimal target that conforms to the dataset's features through pre-training, and then train the network accordingly. Fig. S6A shows the optimized target by contrastive learning on the MNIST dataset. It can be seen that compared with the original design in the manuscript, the optimized target does not follow the logical sequence, in contrast, it is aligned with the feature of the dataset. Fig. S6B shows output results and Fig. S6C shows the confusion matrix of the classification accuracy with a new target, the recognition accuracy has been improved to 88.4% (~84.3% with the original target).

2)	Unsupervised learning method

Nevertheless, unsupervised learning can be used for clustering training to design the target. Based on this approach, the network can automatically find potential classification rules in the training set, making outputs of the same type closer and outputs of different types further apart. The method involves adjusting the loss function to a contrastive loss, and the specific calculation process is as follows: First, the output of the light field is normalized:

$E_{\text{norm}}\text{=}\frac{E}{\left\|E\right\|}$ (S9)

Where ***E*** is the output light field of the ST-AONN forward propagation model, then its similarity ***S*** is calculated:

$S=E_{\text{norm}}E_{\text{norm}}^{T}$ (S10)

The similarity matrix is scaled, where *t* is the temperature parameter, set to 0.1 during training:

$S'\text{=}\frac{S}{\text{t}}$ (S11)

Next, the positive sample mask matrix *M^+^* and the negative sample mask matrix *M^-^* are calculated separately, using the following formulas:

$\text{M}^{\text{+}}\text{=}\left\{\begin{matrix} 1 & \text{if labels}\left[\text{i}\right]\text{=labels}\left[\text{j}\right] \\ 0 & \text{if labels}\left[\text{i}\right]\neq\text{labels}\left[\text{j}\right] \end{matrix}\right.$ (S12)

$\text{M}^{-}\text{=1-M}^{+}$ (S13)

This formula marks sample pairs with the same label as 1 and sample pairs with different labels as 0. The positive sample loss function $F_{loss}^{+}$ and the negative sample loss function $F_{loss}^{-}$ are expressed as:

$\text{F}_{loss}^{+}=-\text{log}\left(\frac{\sum_{i=1}^{n} \text{exp}(S')\text{M}^{+}}{\sum_{i=1}^{n} \text{M}^{+}}\right)$ (S14)

$\text{F}_{loss}^{+}=-\text{log}\left(\sum_{i=1}^{n} \text{exp}(S')\text{M}^{-}\right)$ (S15)

Then the total sample loss function is expressed as:

$\text{F}_{loss}\text{=F}_{loss}^{+}+\text{F}_{loss}^{-}$ (S16)

Based on this loss function, the network can be trained without designing the output target. The training objective is to make the similarity of outputs of the same type higher and the similarity of outputs of different types lower. No human intervention is involved in the target design process. After the training, the target waveform of each class can be obtained, as shown in Fig. S7A.

The test set then is fed into ST-AONN to obtain corresponding outputs, which are subjected to similarity analysis with the target waveforms of all classes. The class with the highest similarity is taken as the predicted result. Fig. S7B shows the confusion matrix of the test set, the recognition accuracy has been improved to 90.2% this time. It can be seen that the accuracy is now comparable with classical AONN with only spatial information.

3)	Increasing the scale of the network

In addition to the above methods, the performance of ST-AONN can also be improved by simply expanding its scale. Specifically, the performance of the network can be improved by increasing the number of computing neurons, that is, the number of diffraction pixels. Generally, there are two main ways to achieve this: increasing the number of diffraction layers and the number of diffraction units on each diffraction layer. The simulation results show that increasing the number of diffraction layers in the ST-AONN from 5 to 10 boosts the network’s recognition accuracy on the MNIST handwritten digit dataset to 89.1%. Similarly, increasing the network resolution from 200×200 to 400×400 pixels raise the accuracy to 88.3%.

Supplementary Note 8. Spatiotemporal light field structure evolution in ST-AONN with 5 diffraction layers

Fig. S8 is a schematic diagram of the gradual evolution of the spatiotemporal light field structure after passing through 5 diffraction layers in ST-AONN. The training target of the network is designed according to the method of Fig. 3A in the manuscript. For incident spatial light fields with different labels, ST-AONN distributes the intensity of the output spatiotemporal fields at different time positions, so its time domain output has different pulse peak delay characteristics. Take the spatial distribution of the input field of the handwritten digits “0” and “9” in Figs. S8A and 8B as example: when the input is “0”, after passing through 5 diffraction layers, most of the energy of the output space-time field will be concentrated on the left side of the time origin (the peak position of the input space-time field is the time origin); when the input is “9”, after passing through 5 diffraction layers, most of the energy of the output space-time field will be concentrated on the right side of the time origin. In the case of multiple classifications, the output field time axis is divided into more time delay regions, corresponding to different labels, as shown in the handwritten digit classifier study in the manuscript. Fig. S8C shows the pulse shaping after passing through each diffraction layer in ST-AONN when the input is “0” and “9” respectively. It shows that although the input pulse shapes of the two cases are identical, the formed pulse shapes after passing through the network are significantly different and gradually change towards the target design.

Supplementary Note 9. Classification study based on the MNIST fashion dataset

In addition to the classification study of handwritten digits in the manuscript, we also conducted research on the MNIST fashion dataset based on the same network design, and the numerical results are shown in Fig. S9. Fig. S9A shows 10 spatial inputs (with different labels) from the test dataset and the corresponding output results in the time domain. It can be seen that the time position of the strongest peak in the output pulse can be used as a classification criterion. Fig. S9B shows the phase distributions of the 5 diffraction layers in the ST-AONN after training. Fig. S9C shows the confusion matrix of the fashion classifier, with a simulation accuracy of 71.6%.

Supplementary Note 10. The digital processing and scene reconstruction of the ST-AONN lidar

Fig. S10 shows the digital processing and scene reconstruction of the ST-AONN lidar. In the experiment, a single-pixel PD was used to capture the temporal output waveform. The signal within each time window was integrated in the time domain to generate a feature value corresponding to each detected targets. In this work, three targets were measured, corresponding to the time windows (t₁–t₂), (t_2_–t_3_), and (t_3_–t_4_), which reflect their respective distances from the lidar system. Each feature value was then compared to V_min_ and V_max_—the feature values obtained when the object was positioned at the far-right and far-left of the measurement range, respectively. The relative position of a feature value between V_min_ and V_max_ corresponds to the actual spatial location of the corresponding target within the detracted scene. Finally, 3D scene reconstruction was achieved based on the resolved positions of all detected objects.

Supplementary Note 11. ST-AONN OAM demultiplexer for high-speed optical communication applications

Fig. S11 presents the demodulation analysis of spatially multiplexed OAM light fields. Our investigation encompassed 7 distinct OAM modes, including fundamental states (*l* = 1, 2, 3) and their multiplexed combinations. To ensure compatibility with high-speed ST-AONN systems, we implemented an encoding scheme that projects different input OAM modes into corresponding output pulse intensities, as shown in Fig. S11A. The network architecture, comprising two diffraction layers, was trained through iterative exposure to all 7 OAM modes using the mean squared error (MSE) method^10,11^, its mathematical expression is:

$Loss_{MSE}=\frac{1}{n}\sum_{i=1}^{n} \left(y_{i}-\hat{y}_{i}\right)^{2}$ (S17)

and the energy of a single pulse is the classification criterion. Fig. S11B displays the trained phase profiles of both diffraction layers. Fig. S11C presents the simulated temporal output pulses for the seven multiplexed OAM modes, demonstrating consistent FWHM durations of ~200 fs. This OAM demultiplexer is compatible with the high-speed ST-AONN system, which can break through the speed limit of the traditional spatial demultiplexing method. Based on the high-speed OAM pulse laser source, which will remove the input speed limitation of two-dimensional light fields, the operation speed of the ST-AONN demultiplexer can reach the GHz level, which is of great significance to the development of high-speed optical communications.

Supplementary Note 12. Implementation of high-speed ST-AONN

There are two approaches to make the high-speed ST-AONN physically constructible. One way is to change the input light source of ST-AONN, ultrafast lasers with a wider spectrum can be used to improve the modulation ability of the network, or broaden the input pulses to ns level that can be directly detected. However, the modulation capability of the network is not only limited by the spectral width of the laser source, but also by the spatial resolution and number of diffraction layers. Improving the modulation capability of the network by using lasers with wider spectra is limited and cannot modulate the pulses to a level that can be directly detected. Meanwhile, ultrafast lasers with wider spectra mean shorter laser pulses. Pulses below 100 fs will produce uncontrollable dispersion chirps and even nonlinear effects when propagating in air and optical components, which are difficult to model and analyze. In addition, extending the input pulse from sub-ps to the ns level requires the introduction of extremely large chirps, which have problems with controllability and stability. Moreover, even if the incident field is successfully expanded to a ns pulse, the chirp introduced by ST-AONN will be difficult to modulate the pulse due to its inherent large chirp.

Another way to achieve high-speed ST-AONN is to improve the network target design. Due to the limited bandwidth of PDs, its detection temporal resolution is insufficient, resulting in the inability to accurately detect ps-level pulses. To overcome this problem, the target design method in the manuscript is adopted, that is, different types of input spatial distributions correspond to different output pulse energies. This design does not require the measurement of pulse form and time delay, and the broadening effect caused by direct detection will not affect the energy relationship between different pulses. Through this design, the physical construction of high-speed ST-AONN can be achieved.

Supplementary Note 13. High-speed ST-AONN

Due to the constraints in detection speed, the interferometer-based temporal measurement method proposed above cannot meet the network's high-speed requirements. To address this, a direct measurement approach using high-speed PD was adopted. Due to bandwidth limitations, conventional PDs (tens of GHz) have insufficient temporal resolution when measuring ultrafast pulses (ps level and below), thus compromising accurate waveform characterization. Therefore, a new network design was adopted, where the output pulses are distinguished by their peak intensities rather than the precise temporal shape. With this new target, the bandwidth limitation of PDs will not compromise the relative pulse intensity relationship between outputs, thus enabling the construction of high-speed ST-AONN. Fig. S12A is the schematic diagram of the high-speed ST-AONN setup. The same fs laser as in the spatiotemporal light field measurement system is adopted, and a DMD is used to generate high-speed switchable 2D spatial inputs. A single-pixel PD and a high-speed oscilloscope are used to detect the temporal signal of the outputs. See the “Materials and Methods” section for details of the setup.

To validate the potential practical use of high-speed ST-AONN, we constructed a dataset emulating the altitude and attitude of aircraft during takeoff and landing and performed motion tasks with it. Fig. S12B shows the 5 classifications in the aircraft motion dataset, where the aircraft differ in altitude and pitch angle. The “Materials and Methods” section provides a detailed description of the dataset. Fig. S12C is the phase distribution obtained from training. Figs. S12D and 12E show the simulation and experimental output results of 5 inputs in the test dataset with different labels. It can be seen that there is a magnitude difference in the pulse width between the simulation and experimental results due to the bandwidth limitation of PD. However, in terms of the pulse intensity relationship, the experimental and simulation results are in good agreement and can be used for classification tasks. Figs. S12F and 12G are the confusion matrixes of the simulations and the experiments, with classification accuracies of 91.6% and 83.6% respectively. The performance differences between high-speed ST-AONN and interferometer-based ST-AONN were compared, and the influence of spatial asymmetry on the network performance was studied.

In addition, we monitored the takeoff and landing process of the aircraft based on the high-speed ST-AONN, and the dynamics and transient behaviors of the system during high-speed operation were analyzed. Since the bandwidth of the PD used in the experiment is much higher than the laser repetition rate and the DMD switching rate, the network operation speed will not be limited by the detection but by the input switching speed. Fig. S12H shows the time domain detection results when DMD continuously switches input images at the highest speed. The input images depict the aircraft landing process. Since the laser repetition rate is higher than the DMD switching rate, multiple pulses are included in one input period (~92 μs), and the network operation speed is the switching speed of the DMD (~17 kHz). In addition, due to the flipping of the DMD micromirrors and the resulting mechanical vibration, the input field will produce unpredictable errors during the switching process (~20 μs). The operating speed of this system is limited by the speed of input which can be improved if faster modulators are used^12-14^.

DMD is composed of micromirrors arranged in an array, the deflection direction of the micromirrors represents the on or off state, as shown in Fig. S13A. When the micromirrors are in the on-state, the reflected light in the corresponding area will enter the system, and when the micromirrors are in the off-state, the light will be reflected in other directions. Therefore, the micromirror's on/off state distribution can generate and switch the spatial target light field. In addition, the micromirrors of the DMD deflect along their diagonal direction. To ensure the horizontal light field output, the DMD chip needs to be placed at 45°. Moreover, the micromirror array of the DMD constitutes a 1D blazed grating structure, as shown in Fig. S13B, which will produce dispersion on the light field. The relationship between the grating period (*d*) and the side length (*l*) of the micromirror can be expressed as $d=\sqrt{2}l/2$.

Fig. S13C shows the angle change of a single micromirror of the DMD during on-off switching. It can be seen that when the switching electrical signal reaches the micromirror, the deflection angle of the micromirror changes (from -12° to 12°, or from 12° to -12°), and this process is completed in about 5 μs. However, due to the mechanical vibration caused by switching, the micromirror will not stabilize immediately. After 10 μs of vibration, the micromirror gradually stabilizes. In addition, due to the large number of micromirrors contained in the DMD, there are communication delays and differences in the preparation of micromirrors, the process of switching to stabilization of the DMD takes slightly longer than that of a single micromirror. Fig. S13D shows the time domain outputs of the ST-AONN with DMD as input. Figs. S13E and 13F are the enlarged views of the corresponding switching areas in Fig. S13D, respectively. It can be seen that the process of closing the previous input image is faster than the process of generating the next input image. This is because when the image is closed, the impact of the micromirror vibration does not enter the system, while when the image is generated, the vibration will affect the input light field. Therefore, in the time domain output, the impact of the vibration can be seen when the signal rises. The switching processes between different images are all ~20 μs.

Supplementary Note 14. Performance differences between high-speed ST-AONN and interferometer-based ST-AONN

We compared the recognition capabilities of high-speed ST-AONN and interferometer-based ST-AONN on aircraft motion datasets, as shown in Fig. S14. Fig. S14A displays five spatially distinct inputs (each with different labels) along with their corresponding time-domain output waveforms. Fig. S14B presents the confusion matrix, demonstrating a recognition accuracy of 93.0%. The simulation accuracy based on high-speed ST-AONN is 91.6%. The results show that there is no significant accuracy compromise for high-speed ST-AONN, because the only difference between the two networks is the detection method, while the network structure is completely identical.

In addition, we compared the system size and temporal performance of high-speed ST-AONN and interferometer-based ST-AONN networks. In terms of system size, the high-speed ST-AONN substantially reduces both physical scale and complexity by eliminating the interferometric setup. Their size is primarily determined by the spatiotemporal modules (gratings, lenses, modulators) and the propagation distances between them. These components are inherently easier to miniaturize, thus offering greater potential for on-chip integration. In terms of temporal performance, the interferometer-based ST-AONN enables complete characterization of the output spatiotemporal light field structure and achieves superior temporal resolution compared to the single-pixel PD detection. The temporal resolution of an interferometer-based ST-AONN depends the accuracy of the time delay and the pulse width of the reference beam; for the experimental setup in this work, its temporal resolution is approximately 100 fs. In contrast, the temporal resolution of a high-speed ST-AONN depends on the bandwidth of the PD and oscilloscope, and is approximately 100 ps.

Supplementary Note 15. Effect of spatial asymmetry on ST-AONN

We have designed two distinct datasets: one featuring motion patterns along the y-axis(modulated) and another along the x-axis(unmodulated), as shown in Figs. S15A and 15B. We trained two datasets based on single-layer ST-AONN (input spatially dispersed frequencies along the x-axis), and obtained simulation test prediction accuracies of 96.6% and 92.4%, respectively. It can be seen that the recognition accuracy of ST-AONN for datasets with main features along the modulated axis (y) is only slightly higher than that for datasets with main features along the unmodulated axis (x). Figs. S15C and 15D show the light field distribution in the k_y_-ω plane of the y-axis and y-axis moving datasets, as well as the output spatiotemporal light field structures and time-domain waveforms, respectively. It can be seen that, after dispersion, the group of inputs with features varying along the y-axis exhibits significantly greater variation than the group with features along the x-axis. And we believe this is where the recognition difference originated.

Nevertheless, the example we put here is a relatively simple task, when it comes to more complex inputs, the fact that one spatial dimension has not been modulated may cause more important drop in performance. In this case, one should preprocess the input signal in advance accordingly. For example, by rotating the input light field signal, feature detection along orthogonal spatial dimensions can be achieved. This method ensures complete feature coverage of the input dataset and fully utilizes the recognition capability of the ST-AONN architecture.

Supplementary Note 16. System energy efficiency analysis

Taking the single-layer ST-AONN lidar configuration with 200×200 modulation units in Fig. 4c as an example, the system achieves a frame rate of 76 Mfps, performing 3.04 trillion operations per second (200*200*7.6*10^7^) in its forward model^15,16^. For 8-bit image processing, this corresponds to a computational throughput of 778.3 TOPS (tera-operations per second) (3.04*10^12^*2^8^). When extended to a 5-layer architecture, the system's processing capacity increases to 3891.2 TOPS. Experimentally, with a laser input power of 1W, the theoretical computational energy efficiency exceeded 10³ TOPS/W. When accounting for the system power consumption—including the laser source (~400W), SLM (~20W), single-pixel PD (~1W), totaling approximately 500W—the overall system energy efficiency reaches about 7.8 TOPS/W.

As shown in Table S1, this energy efficiency demonstrates advantages over conventional electronic computing platforms, such as HUAWEI Ascend 910 (1.65 TOPS/W)^17^, NVIDIA Tesla T4 (1.85 TOPS/W)^18^, and Google TPU (4.65 TOPS/W)^19^. However, it remains lower than some specialized optical computing systems, including optical vector convolutional accelerator (11 TOPS/W)^20^, Large-scale photonic chiplet Taichi (160 TOPS/W)^21^. This difference primarily stems from the substantially higher energy consumption of fs laser sources compared to continuous-wave or low-power pulsed lasers.

**Fig. S1. Schematic of the ST-AONN.** ST-AONN is a 4*f* configuration composed of grating-cylindrical lens pairs and diffraction layers, the diffraction layer can be a single layer or multiple layers.

**Fig. S2. Schematic diagram of measuring the target spatiotemporal field.** The reference pulse can measure and reconstruct the spatiotemporal distribution of the target light field by temporal scanning.

**Fig. S3. Matrix dimensions in ST-AONN numerical training.** (**A**) Input light field and input image. (**B**) Diffraction layers. (**C**) Output mask and output light field.

**Fig. S4.** **Simulation results of the target design based on the number of peaks.** (**A**) Target design based on peak number. (**B**) Test dataset with different labels and the corresponding output results. (**C**) Simulated confusion matrix.

**Fig. S5. The simulation results of a space-only diffraction AONN.** (**A**) The target design of diffraction AONN. (**B**) Confusion matrix of the simulated digit classifier.

**Fig. S6. Improving the performance of ST-AONN by appropriate target design.** (**A**) Target design conforms to the features of the dataset through pre-training. (**B**) Test dataset with different labels and the corresponding output results. (**C**) Simulated confusion matrix.

**Fig. S7. Unsupervised learning method based on clustering training.** (**A**) The training process of unsupervised learning and the targets of classification. (**B**) Confusion matrix of the simulated digit classifier based on unsupervised learning.

**Fig. S8. Schematic diagram of spatiotemporal light field changing in 5-layer ST-AONN.** (**A**) When the input is “0”, the field intensity gradually moves to the left of the time axis after 5-layer ST-AONN modulation. (**B**) When the input is “9”, the field intensity gradually moves to the right of the time axis after 5-layer ST-AONN modulation. (**C**) The temporal profiles evolution after passing through each diffraction layer for input digits “0” and “9”. The central figures are the phase distribution of the 5 diffraction layers obtained from training.

**Fig. S9. Classification studies of MNIST fashion database.** (**A**) 10 inputs in the MNIST test dataset with different labels and the corresponding output results in the time domain. (**B**) Phase distributions of 5 diffraction layers. (**C**) Confusion matrix of the simulated digit classifier.

**Fig. S10. The digital processing and scene reconstruction of the ST-AONN lidar.**

**Fig. S11. ST-AONN OAM demultiplexer.** (**A**) 7 input OAM with different multiplexed combinations and the corresponding output results. (B) The phase distribution of the 2 diffraction layers obtained from training. (C) Simulated temporal output pulses for the seven multiplexed OAM modes.

**Fig. S12. High-speed ST-AONN.** (**A**) Schematic diagram of high-speed ST-AONN setup. (**B**) Examples in aircraft motion dataset with 5 labels (Statues 1~5). (**C**) The phase distribution loaded on the SLM. (**D**) The simulation results of the 5 test datasets with different labels. The FWHMs of the simulated output pulses are ~200 fs. (**E**) The experiment results of the 5 test datasets and the FWHMs of the experiment output pulses are ~0.2 ns. (**F**) Simulated confusion matrix of 500 test datasets in the aircraft motion dataset. (**G**) Experimental confusion matrix of the same test dataset. (**H**) The time domain detection results of the emulating aircraft landing process. The motion is achieved by continuously switching the input images of the DMD at the highest speed.

**Fig. S13. The structure and switching of DMD.** (**A**) Structure of one micromirror of the DMD. The micromirrors can be individually rotated by ±12° to an on or off state. (**B**) Schematic diagram of the micromirror array structure of DMD. The deflection of the micromirrors is along their diagonals, similar to a 1D-blazed grating structure. (**C**) Angular variation of a single micromirror during DMD switching. (**D**) The time domain output results of the ST-AONN with DMD as input. (**E**) and (**F**) are the enlarged views of the corresponding switching areas in (D), respectively.

**Fig. S14. Classification studies of aircraft motion dataset based on the interferometer-based ST-AONN.** (**A**) 5 input digits in the aircraft motion dataset with different labels and the corresponding output results. (**B**) Confusion matrix of the simulated test results.

**Fig. S15.** **Effect of spatial asymmetry on the performance of ST-AONN.** Datasets with patterns moving along the (**A**) y-axis and (**B**) x-axis. (**C**) and (**D**) are the spatiotemporal light field structures and time-domain waveform outputs of the y-axis and x-axis moving datasets.

| Technology | Theoretical energy efficiency | Works |
| --- | --- | --- |
| HUAWEI Ascend 910 | 1.65 | Ref. ^17^ |
| NVIDIA Tesla T4 | 1.85 | Ref. ^18^ |
| Google TPU | 4.65 | Ref. ^19^ |
| Optical vector convolutional accelerator | 11 | Ref. ^20^ |
| Large-scale photonic chiplet Taichi | 160 | Ref. ^21^ |
| ST-AONN | 7.8 | This work |

**Table S1.** **Energy efficiency comparison of partial different optical computing architecture and electronic hardware**

**Captions for Supplemental Movie:**

**Supplementary Move 1.** (**A**) Lidar input scenario for multiple targets. (**B**) Real-time temporal output on the oscilloscope. (**C**) Real-time recovery of multi-target motion.

References

1. Chong, A. Wan, C. H. Chen, J. & Zhan, Q. Generation of spatiotemporal optical vortices with controllable transverse orbital angular momentum. *Nat. Photonics* **14**, 350-354 (2020).
2. Huang, S. Wang, P. Shen, X. & Liu, J. Properties of the generation and propagation of spatiotemporal optical vortices. *Opt. Express* **29**, 26995-27003 (2021).
3. Wefers, M. M. & Nelson, K. A. Space-Time Profiles of Shaped Ultrafast Optical Waveforms. *IEEE J. Quantum Elect.* **32**, 161-172 (1996).
4. Hancock, S. W. Zahenpour, S. Goffin, A. & Milchberg, H. M. Free-space propagation of spatiotemporal optical vortices. *Optica* **6**, 1547-1553 (2019).
5. Martinez, O. E. Grating and prism compressors in the case of finite beam size. *Optical Society of America* **3**, 929-934 (1986).
6. Weiner, A. M. Femtosecond pulse shaping using spatial light modulators. *Rev. Sci. Instrument.* **71**, 1929–1960 (2000).
7. Kondakci, H. K. & Abouraddy, A. F. Diffraction-free space-time light sheets. *Nat. Photon.* **11**, 733–740 (2017).
8. Weiner, A. M. Ultrafast optical pulse shaping: A tutorial review. *Opt. Commun.* **284**, 3669-3692 (2011).
9. Kingma, D. P. & Ba, B. L. Adam: A method for stochastic optimization. *arXiv* **1412**, 6980 (2014).
10. Tian, Y. Su, D. Lauria, S. & Liu, X. Recent advances on loss functions in deep learning for computer vision. *Neurocomputing* **497**, 129-158 (2022).
11. Pan, Z. Yu, W. Wang, B. Xie, H. Sheng, V. S. Lei, J. & Kwong, S. Loss functions of generative adversarial networks (GANs): Opportunities and challenges. *IEEE Transactions on Emerging Topics in Computational Intelligence* **4**, 500-522 (2020).
12. Liu, K. Ye, C. R. Khan, S. & Sorger, V. J. Review and perspective on ultrafast wavelength‐size electro‐optic modulators. *Laser Photonics Rev.* **9**, 172-194 (2015).
13. Yang, Y. Forbes, A. & Cao, L. A review of liquid crystal spatial light modulators: devices and applications. *Opto-Electronic Science* **2**, 230026-1 (2023).
14. Bartlett, T. A. McDonald, W. C. Hall, J. N. Oden, P. I. Doane, D. Ketchum, R. S. & Byrum, T. Recent advances in the development of the Texas Instruments phase-only microelectromechanical systems (MEMS) spatial light modulator. *Emerging Digital Micromirror Device Based Systems and Applications XIII* **11698**, 103-116 (2021).
15. Fu, T. Zhang, J. Sun, R. et al. Optical neural networks: progress and challenges. *Light-Sci Appl.* **13**, 263 (2024).
16. Duan, Z. Chen, H. Lin, X. Optical multi-task learning using multi-wavelength diffractive deep neural networks. *Nanophotonics* **12**, 893-903 (2023).
17. HUAWEI. Ascend 910 at URL: https://www.actfornet.com/products/intelligent-computing/atlas/huawei-ai/ai-chips/Ascend_910 /features (2024).
18. NVIDIA. T4 tensor core datasheet at URL: https://www.nvidia.com/en-us/ data-center/tesla-t4/ (2024).
19. Jouppi, N. P. et al. In Proc. ACM/IEEE 44th Annual International Symposium on Computer Architecture (ISCA), 1–12, (2017).
20. Xu, X. Tan, M. Corcoran, B. et al. 11 TOPS photonic convolutional accelerator for optical neural networks. *Nature* **589**, 44-51 (2021).
21. Xu, Z. Zhou. T. Ma. M. et al. Large-scale photonic chiplet Taichi empowers 160-TOPS/W artificial general intelligence. *Science* **384**, 202-209 (2024).
